# A new approach to solve the Brachistochrone problem by constructing a lattice unit cell

**DOI:** 10.1016/j.heliyon.2022.e11994

**Published:** 2022-12-08

**Authors:** Esam H. Abdul-Hafidh

**Affiliations:** Physics Department, Faculty of Science at Taibah University-Yanbu, King Khalid Rd. Al Amoedi, 46423, Yanbu El-Bahr, 51000, Saudi Arabia

**Keywords:** Brachistochrone, Computational methods, Condensed matter physics, Lattice unit cell, Cycloid

## Abstract

A simple and general new approach to solve the Brachistochrone problem is presented in this paper. The Brachistochrone problem is concerned with finding the shortest time trajectory of a particlesliding on a frictionless path under gravity. The problem is solved in this project using a solid-state physics mechanism of building a lattice by a unit cell of a suitable lattice parameter and a transformation operator. This problem was solved analytically centuries ago by many scientists. To the author's knowledge, the approach considered here was not used before. The method clearly shows that the Brachistchrone is just a two-dimensional lattice with a parameter and a transformation angle that depend on the size of the trajectory. It has been found that the shortest time track is a cycloid, which is a curve that lies between a straight line and a circle. Thefindings of this work were compared to the exact results found previously and were found to be within an infinitesimally negligible margin of error.

## 1 Introduction

More than three centuries have passed since the Brachistochrone (shortest time) problem was solved [1, 2, 3, 4, 5, 6, 7, 8, 9, 10]. The solution was made manifest by many scientists by then, but the credit to solve it was given toJohan Bernoulli in 1697, who published the challenge to his fellows [5].

It was proven that a particle sliding under gravity from point A to point B in a vertical plane traverses in the shortest time if it moves over a cycloidal trajectory [6]. Johan Bernoulli used the optical Fermat concept to solve the problem [6]. Using the calculus of variations, Euler addressed the method that gave a rigorous proof of the Brachistochrone [1]. The form of the trajectory is not affected for a rolling uniform sphere [6].

Many researchers worked on generalizations of the Brachistochrone in the past few decades. Their research topics and findings fall under five main subjects that answer the questions: What are the shapes of the trajectories (1) if friction is considered, (2) if the gravitational field is not uniform, (3) if the motion is relativistic, (4) if the motion is constrained to a cylindrical path, and (5) if the motion is under an inverse square law force. All of these cases showed deviations from the cycloidal path [2, 3, 6, 8, 9, 10].

In this project, a numerical approach that uses simple algebra and a solid-state physics mechanism of building a lattice by a unit cell of a suitable lattice parameter and a transformation operator is suggested to solve the original Brachistochrone problem of a point-like particle moving under the force of gravity on a frictionless trajectory.

The method involves finding a point-by-point approximation of the trajectory of the sliding particle by optimizing the lattice parameter, angle and the number of lattice points. The present work handles the original brachistochrone as a discrete model of motion, where any segment of the optimal trajectory is assumed to be optimal. This means that a narrow class of piecewise smooth functions are considered here to solve the frictionless Brachistochrone. Considering dry or viscous friction, the solution to the problem will differ significantly. This paper is organized as follows. The method is presented in Section 2, ending with the equations that will be used to find the trajectory of the Brachistochrone. Section 3 is devoted to a numerical example that shows the comparison between the exact solution to the problem and the prediction of this work. Section 4 contains concluding remarks.

## 2 Methods of calculation

There are many standard solutions to the Brachistochrone problem. The model used here employs the concept of crystal lattice in solid-state physics. The trajectory of the shortest time between points A and B for a particle sliding under gravity in a vertical plane is constructed by a unit cell and a transformation operator in the same way as crystals are built in solid-state physics. The transformation vector is decided by a lattice parameter “*a*” and an angle “*θ*”.

**Figure 1:**
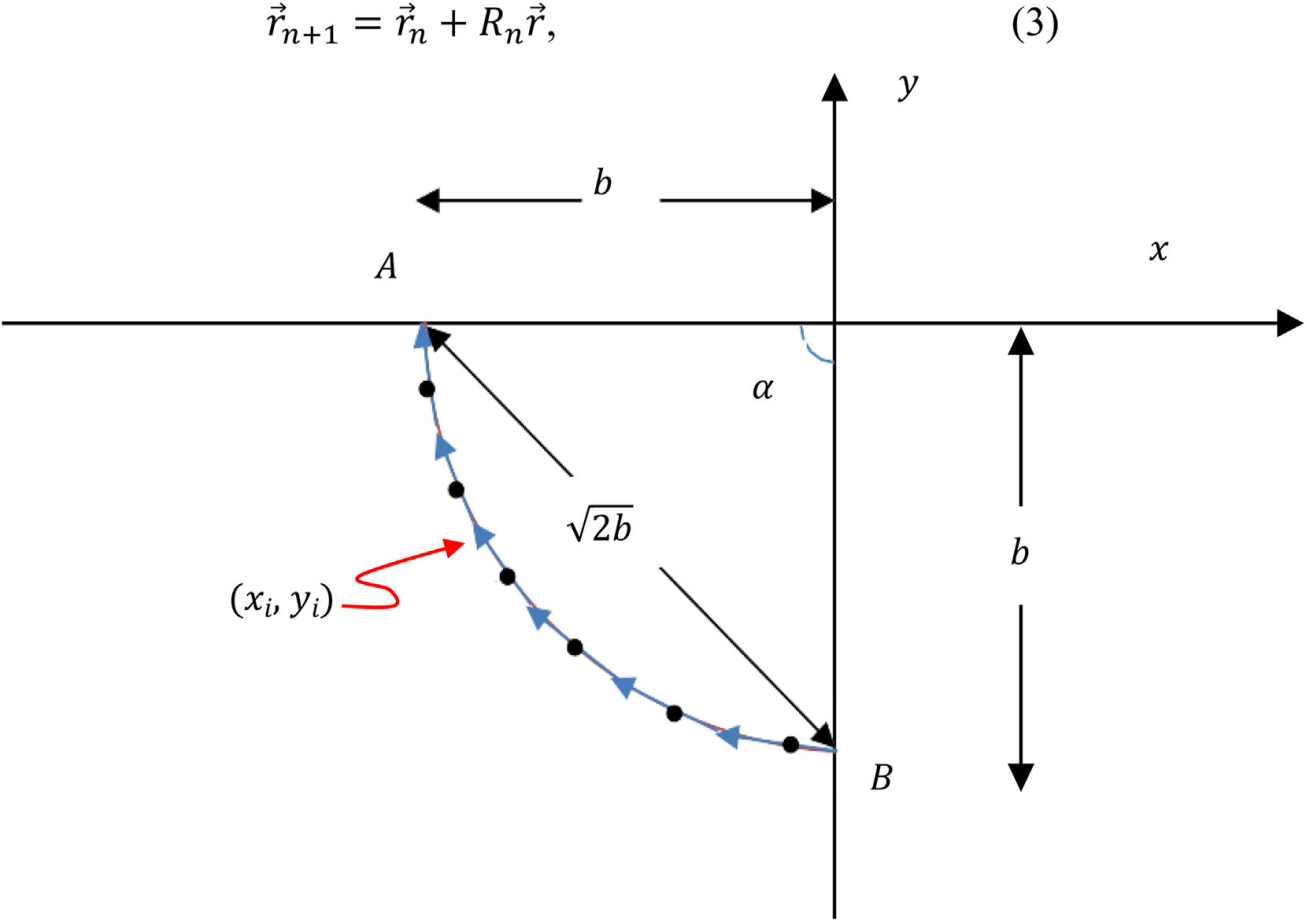
The dots represent the points that form the path of the particle, where the arrows are tangents to a curve that represents the trajectory. All arrows have the same lengths and slopes.

As is known, the shortest distance between two points in *x* − *y* space is the straight line. By analogy, the shortest time is a straight line linking the two points in the *t* − *θ* space, where *θ* is the relative change in direction. i.e., *θ* = *tan*^−1^ (*dy*/*dx*). The *dy*/*dx* represents the slope of the curve in the *x* − *y* plane. For linear *θ* in time, *dθ*/*dt* = *constant*. i.e., at equal time-intervals on the shortest trajectory, (*dy*/*dx*) is a constant value with respect to the previous point. Therefore, the slope of the tangents to the trajectory on the *x* − *y* plane changes equally at fixed times. With the help of Figure 1, the arrows represent the tangents at the dots. Every arrow has the same slope with respect to the head of the previous one.

Having established that, we demonstrate now the algorithm of constructing a lattice using a unit cell. The main ideas of this mechanism are standard and explained in many solid-state physics books, such as the “Introduction to Solid State Physics” by Charles Kittel [11]. The unit cell is composed of one lattice point at the tail of every arrow (vector). This vector has a length equal to the lattice parameter “*a*”.

Starting with the first lattice point at (0,−b), every lattice point is created by the rotation matrix $R_{n}$ that rotates the vector $\vec{r}$ by an angle nθ, where n=1,2,3,… The first lattice point is a vector that can be represented by the column vector:

(1) $${\vec{r}}_{0}=\left(\begin{array}{c} 0\\ -b \end{array}\right)$$

and the vector:

(2) $$\vec{r}=\left(\begin{array}{c} -a\;\cos\;\theta \\ a\;\sin\;\theta \end{array}\right)\text{.}$$

The operator $R_{n}$ is a clockwise rotation matrix with an angle nθ; $R_{n}=\left(\begin{array}{cc} \cos\;n\theta & \sin\;n\theta \\ -\sin\;n\theta & \cos\;n\theta \end{array}\right)$.

Therefore, to create all lattice points (the set of points that form the trajectory), the following vector is used:

(3) $${\vec{r}}_{n+1}={\vec{r}}_{n}+R_{n}\vec{r}\text{,}$$

${\vec{r}}_{n}$ is the *n*th vector extending from the origin to a distinct lattice point. The heads of the arrows in Figure 1 represent the lattice points. The desired lattice that forms the trajectory is configured by a and θ and the number of lattice points N. To form a smooth, continuous (large N) and accurate trajectory that compares to the exact cycloid by an infinitesimally negligible error, a and θ need to approach zero. The deviation from the cycloidal trajectory is larger for small N, bigger a and θ. From Eqs. (1), (2), and (3), $x_{0}=0$, $y_{0}=-b\text{.}$ $x_{1}=-a\;\cos\;\theta$, $y_{1}=a\;\sin\;\theta -b$. $x_{2}=-a\;\cos\;\theta -a\;\cos\;2\theta$, $y_{2}=a\;\sin\;\theta +a\;\sin\;2\theta -b$. Therefore,

(4) $$x_{N}=-a\sum_{n=1}^{N}\cos\;n\theta ,y_{N}=a\sum_{n=1}^{N}\sin\;n\theta -b$$

In Figure 1, the distance AB is equal to $\sqrt{2}b$ whenever α=π/2 (subcritical geometry) [8].

**Figure 2:**
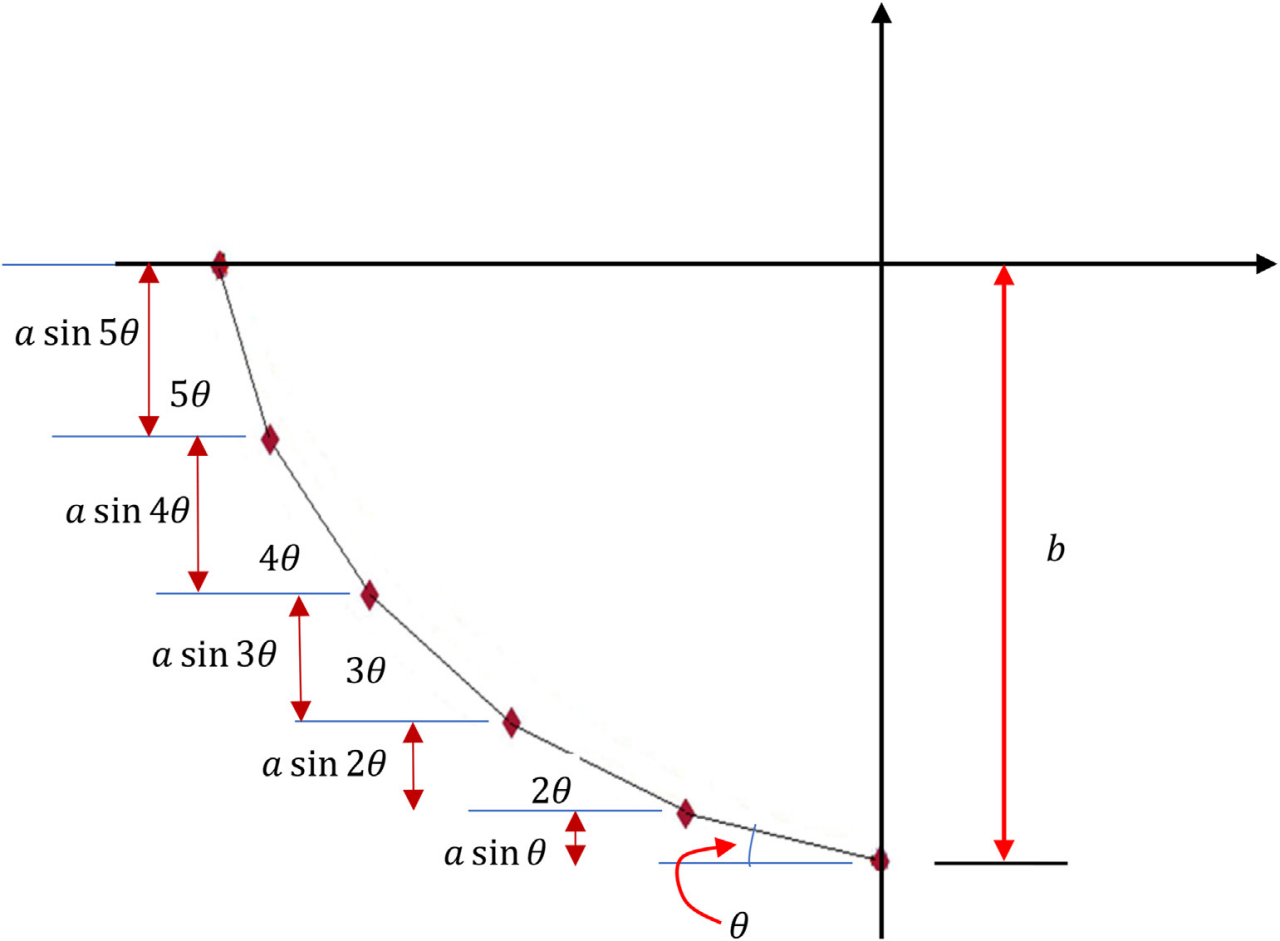
Lattice points and the relationship between b and a.

For a given a and b, the values of θ and N need to be determined. With the help of Figure 2, it is clear that:

(5) $$\theta =\frac{\alpha }{N}=\frac{\pi }{2N}$$

Also, a and b are related via:

(6) $$b=a\sum_{n=1}^{N-1}\sin\;n\theta \text{.}$$

By Using Lagrange's identity, the summation of Eq. (6) is explicitly given by:

(7) $$\sum_{n=1}^{N-1}\sin\;n\theta =\frac{1}{2}\cot\left(\frac{1}{2}\theta \right)-\frac{1}{2}\frac{\cos\left(N-\frac{1}{2}\right)\theta }{\sin\left(\frac{1}{2}\theta \right)}\text{.}$$

Therefore, $\frac{b}{a}=\frac{1}{2}\cot\left(\frac{1}{2}\theta \right)-\frac{1}{2}\frac{\cos\left(N-\frac{1}{2}\right)\theta }{\sin\left(\frac{1}{2}\theta \right)}$ [12]. Substituting the value of θ in Eq. (5) into Eq. (7), gives:

$$\frac{b}{a}=\frac{1}{2}\cot\left(\frac{\pi }{4N}\right)-\frac{1}{2}\frac{\cos\left(N-\frac{1}{2}\right)\frac{\pi }{2N}}{\sin\left(\frac{\pi }{4N}\right)}$$

This equation reduces to:

(8) $$\frac{b}{a}=\frac{1}{2}\cot\left(\frac{\pi }{4N}\right)-\frac{1}{2}$$

This equation can be solved by choosing a large number of lattice points $N(\geq 10^{3}$ to give a continuous trajectory. θ is found from Eq. (5). For an arbitrary value of b, “a” is calculated from Eq. (8). At this stage, an example can demonstrate the procedure given in section (2), where the coordinates of every lattice point can be exactly defined and therefore the trajectory can be drawn, for given arbitrary values of a and b.

## 3 Results and discussion

Using the method explained in Section 2 and a customized MATLAB code (See Appendix), the optimum point-by-point trajectory of a particle sliding down on a frictionless path under gravity is found as follows: The coordinates of first point are (0,−b).

To draw a trajectory, the number of lattice points N can be arbitrarily chosen. For N=6, the values of the lattice parameter a and the transformation angle θ are found with the help of Eqs. (1) and (8) as follows:

θ=α/N=π/12=15°. $b=a\sum_{n=1}^{N-1}\sin\;n\theta$, for any value of b. $\sum_{n=1}^{5}\sin\;n\theta =\frac{1}{2}\cot\left(\frac{\pi }{24}\right)-\frac{1}{2}\frac{\cos\left(\frac{11\pi }{24}\right)}{\sin\left(\frac{\pi }{24}\right)}=3.297877.\;Choosing\;b=-6.85958,the\;lattice\;parameter\;is\;a=-\frac{6.85958}{3.297877}=-2.08\;units$. With these values of a=2.08unitsandθ=15°(π/12radians), there are 6 lattice points only and the values of the coordinates of these lattice points are calculated using Eqs. (1) and (4). $x_{0}=0,y_{0}=-6.85958.x_{1}=-a\;\cos\;\theta =-2.08\;\cos\;15=-2.00913$, $y_{1}=a\;\sin\;\theta -b=2.08\;\sin\;15-6.85958=-6.32124\text{.}$ The other values are calculated as shown in Table 1:

**Table 1:** Coordinates of the Lattice points generated by the preset method for N=6.

*x*	*y*
0	−6.85958
−2.00913	−6.32124
−3.81046	−5.28124
−5.28124	−3.81046
−6.32124	−2.00913
−6.85958	0

**Figure 3:**
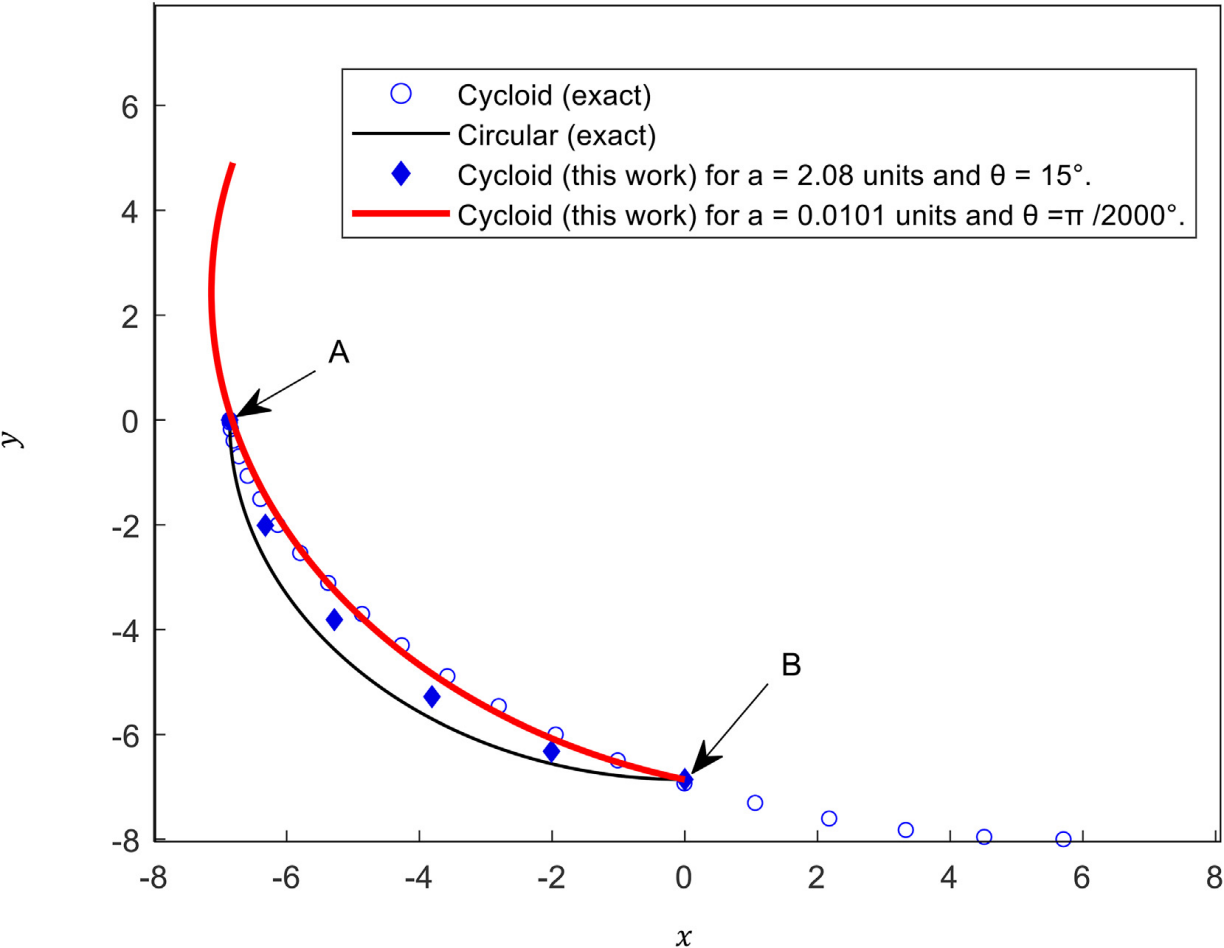
The trajectories of a point-like mass sliding on frictionless paths under gravity.

From these calculations, it is clear that the particle starts at (0, −6.85958) and ends at (−6.85958, 0). i.e., *b* = 6.85958. The data of Table 1 are plotted in Figure 3 and denoted by the diamond shaped points. The trajectory is a curve that touches these tangents.

For the sake of comparison, the cycloid that links A and B is drawn. The cycloid is parametrized by the standard equations x=r(ψ−sinψ) and y=r(1−cosψ). Using r=4.0 and ψ from 0 to π radians, the cycloid is given in Figure 2 by the circle-shaped points.

The deviation of the predicted points $\left(a=2.08\;units\;and\;\theta =15{}^{\circ}\left(\frac{\pi }{12}radians\right)\right)$ from cycloidal is due to the large values of “*a*” and “θ”. As “*a*” and “θ” approach zero (Large N), the curve approaches the cycloidal trajectory as it is clear from the thick solid curve in Figure 3,where the best fit gives a=0.0101, $\theta =\pi /2000{}^{\circ}\left(1.57079\times 10^{-3}\;radians\right)$ and N=1000. It is clear from step 35 of the MATLAB code that red thick curve of Figure 3 has been created by 1000 data points. The angle “π/2” over which the particle moves from point A to point B is divided into 1000 parts. i.e., the transformation angle is “π/2000” which is equal to 1.57/1000. To compare the given trajectory, a circular path with radius 6.85958units is drawn and given by the thin solid line. The circular curve has the equation: $y=-\sqrt{6.8595^{2}-x^{2}}$.

The results that predicted the cycloid in the example given above with b=6.85958units can be extended for other values of b and still will reveal a cycloid with the shortest time among other trajectories.

## 4 Conclusion

In summary, we presented a generalized simple and general model that has been capable of predicting the results found by Bernoulli for the Brachistochrone problem, numerically using the solid-state physics mechanism for building a lattice from a unit cell. The unit cell is composed of one lattice point and a vector of length a. The lattice points are created by transforming the vector by a rotation, as described above. As mentioned in the paper, as “a” and “θ” approach zero, the path gets smoother and accurate. The excellent agreement between the predictions of this work and the exact calculations show that the method employed here is powerful, and can be used to solve other problems without going over mathematical complications. However, in case of friction, the track will definitely deviate from cycloid due to energy losses. The current approach is limited to frictionless track. The method of this work can be extended to handle the friction case if the transformation vector (lattice parameter and angle) is modified in such a way to depend on the coefficient of friction.

## Declarations

### Author contribution statement

Esam H. Abdul-Hafidh, Associate Professor: Conceived and designed the experiments; Performed the experiments; Analyzed and interpreted the data; Contributed reagents, materials, analysis tools or data; Wrote the paper.

### Funding statement

This work recived financial and technical support from Taibah University.

### Data availability statement

Data will be made available on request.

### Declaration of interest's statement

The authors declare no conflict of interest.

### Additional information

No additional information is available for this paper.

## Appendix A Supplementary data

Appendix.docx Appendix.docx
