# Supplementary material for: A new approach to solve the Brachistochrone problem by constructing a lattice unit cell
Source: Heliyon. 2022 Dec 8;8(12):e11994. doi: 10.1016/j.heliyon.2022.e11994 (PMC9761604; doi:10.1016/j.heliyon.2022.e11994)
Supplement: Appendix.docx [file mmc1.docx]

*Appendix*

%--------cycloid with r = 4.0--------

clear all: close all:clc

t=0:pi/21:pi;

b=6.86; %arbitrary value

r=4.0;

x=r*(t-sin(t))-b;

y=-r*(1-cos(t));

scatter(x,y,20,'o','b')

%--------Circular Path with a radius b = = 0.7g = 6.859584--------

hold on

x2=-b:0.01:0.0;

r2=b;

y2=-sqrt(r2^2-x2.^2);

plot(x2,y2,'k','LineWidth',1)

%---Cycloid found by the method of this work a = 2.08 and $\theta$ = 15 degrees---

hold on

tt=pi/12;

a=2.08;

x3=0;y3=0;

for N=1:5

x3(N+1)=x3(N)-a*cos(N*tt);

y3(N+1)=y3(N)+a*sin(N*tt);

end

x3=x3;

y3=y3-b;

scatter(x3,y3,30,[0.00 0.00 0.900],'d','filled')

%-Cycloid found by the method of this work a = 0.01015 and $\theta$ = 9/100 degrees--

hold on

Rotation=-0.26;

t=pi/2;

d=1000;

a=0.0151;

x4=0;y4=0;

for N=1:1000;

x4(N+1)=x4(N)-(a)*cos(N*t/d);

y4(N+1)=y4(N)+(a)*sin(N*t/d);

end

x4=x4;

y4=y4;

x5=x4*cos(Rotation)-y4*sin(Rotation);

y5=x4*sin(Rotation)+y4*cos(Rotation)-b;

plot(x5,y5,'r','LineWidth',2)

xlim([-8,8])

ylim([-8,8])
